# Characterization of a new mouse p53 variant: loss-of-function and gain-of-function

**DOI:** 10.1186/1423-0127-21-40

**Published:** 2014-05-08

**Authors:** James Yi-Hsin Chan, Ying-Chuan Chen, Shu-Ting Liu, Wei-Yuan Chou, Ching-Liang Ho, Shih-Ming Huang

**Affiliations:** 1 Department of Microbiology and Immunology, National Defense Medical Center, Taipei 114, Taiwan; 2 Department of Family and Community Medicine, Tri-Service General Hospital, National Defense Medical Center, Taipei 114, Taiwan; 3 Department of Biochemistry, National Defense Medical Center, Taipei 114, Taiwan; 4 Department of Medicine, Division of Hematology/Oncology, Tri-Service General Hospital, National Defense Medical Center, Taipei 114, Taiwan

**Keywords:** p53, p21, Transactivation, Conformation, Dominant negative effect

## Abstract

**Background:**

p53 is a major tumor suppressor that is inactivated in over 50% of human cancer types through either mutation or inactivating interactions with viral or cellular proteins. The uncertainties around the link between p53 status, therapeutic response, and outcome in cancer suggest that additional factors may be involved. p53 isoforms that are generated via the alternative splicing pathway may be promising candidates for further investigation.

**Result:**

In this study, we report one new p53 protein with two internally deleted regions, resulting in one deleted amino acid fragment (from amino acid residues 42 to 89) and one reading frame-shift region (from amino acid residues 90-120) compared to wild-type p53. The functional status of the new p53 protein, which has a defect in its proline-rich and N-terminal DNA-binding domains, was characterized as possessing an intact conformation, exhibiting no transactivation activity, exerting a dominant-negative effect and an interacting with a coactivator with an arginine methyltransferase activity.

**Conclusion:**

Taken together, our findings provide valuable information about the structure and function of p53 for the regulation of transactivation activity and cellular protein-protein interactions. Furthermore, natural p53 isoforms will help us understand the functional roles of the p53 family and potential therapeutics for p53-dependent cancers.

## Background

p53 and two related proteins, p63 and p73, exhibit the three typical domains of a transcription factor: the amino-terminal transactivation domain (TAD), the DNA-binding domain (DBD) and the carboxyl-terminal oligomerization domain (OD) [1-3]. p53 is a major tumor suppressor that is inactivated in over 50% of human cancer types through either mutation or inactivating interactions with viral or cellular proteins [4]. p53 prevents cancer formation by regulating multiple involved pathways in favor of cell cycle arrest or apoptosis [5,6]. p53 is able to trigger the pro-survival or cell death responses that are dependent on the tissue and cell type, the nature and intensity of the stress signal and the extent of cellular damage [7,8]. The uncertainties around the link between p53 status, therapeutic response, and outcome in cancer suggest that additional factors may be involved. p53 isoforms that are generated via the alternative splicing pathway or others may be candidates for further investigation.

p53 isoforms are physiological proteins that are expressed in normal cells and mediated through *TP53* and alternative promoters, splicing sites and/or translational initiation sites [2,3]. In addition to the two p53-related proteins p63 and p73, which share strong structural biochemical and biological homologies, twelve p53 isoforms have been described in human. In mice, six p53 isoforms have been described and result from combinations of three N-terminal p53 isoforms with two different C-terminal isoforms. The N-terminal isoforms lacking the TAD (i.e., Δ40p53, Δ133p53 and Δ160p53) are expected to only act as dominant-negative regulators of p53 activity, whereas the biological functions of the C-terminal p53 isoforms (i.e., p53β and p53γ) remain poorly described and controversial. A recent study indicates a unique role for p53/47 in the p53 pathway and illustrates how a cellular stress can lead to the induction of 14-3-3σ and G2 arrest but not affect G1 progression through expression of a p53 isoform [9]. Hence, p53 isoforms might modulate p53-mediated cell fate outcomes and be key components of the p53-mediated decision not only under basal conditions but also in response to stress.

Approximately half of tumors sustain mutations in the *TP53* gene itself, whereas the other half maintain a wild-type *TP53* gene but acquire other genetic or epigenetic alterations that compromise the p53 response [4,10]. Most of the mutations within the *TP53* gene are missense mutations, resulting in the expression of full-length mutant p53 proteins [4]. Structurally, mutant p53 can be roughly divided into two main classes: those that alter the amino acid residues responsible for forming sequence-specific contacts with DNA (DNA contact mutants) and those that disrupt the global conformation of p53 (conformational or structural mutants) [11]. Functionally, *TP53* mutations result in the loss of wild-type p53 tumor suppressor activities, the acquisition of an ability to suppress the function of the remaining wild-type *TP53* allele via a dominant-negative mechanism, and, at least in some cases, also in wild-type p53-independent gain of oncogenic functions [12].

Here, we report one new p53 protein with two internally deleted regions, resulting in one deleted amino acid fragment (from amino acid residues 42 to 89) and one reading frame-shift region (from amino acid residues 90-120) compared to wild-type p53. It is of interest to characterize the functional status of new p53 variants with defects in their proline-rich domains (PRD) and N-terminal DBD (DNA-binding domain).

## Methods

### Plasmids

New variant and wild-type p53s were synthesized with a mouse 17-day embryo cDNA library (Clontech, USA) as PCR template. Plasmid DNAs encoding wild-type and mutant p53s were cloned into pSG5.HA (hemagglutinin) and Gal4DBD (pM) vectors via a *BamH*I-*Xho*I site. Plasmid DNAs encoding the human estrogen receptor and thyroid receptor have been described previously [13,14]. Reporters for GK1 (Gal4 DNA responsive reporter), pG13-, MMTV(ERE)- and MMTV(TRE)-LUC have been described previously [13,14]. Bacterial expression vectors for glutathione *S*-transferase (GST) fused to various p53 fragments were constructed by inserting the appropriate PCR fragments into the *BamH*I-*Xho*I sites of the pGEX–4 T1 vector.

### Cell culture, transfection and luciferase reporter assays

HeLa and p53^-/-^ MEF cells were cultured in Dulbecco modified Eagle’s medium supplemented with 10% fetal bovine serum and 1% penicillin-streptomycin (Invitrogen, USA). Transient transfections and luciferase assays were performed in 24-well culture dishes as described previously [15]. Luciferase assays were performed using the Promega Luciferase Assay kit. The total DNA used for the reporter analysis was adjusted to 1 μg by adding the necessary amount of empty vector. The luciferase activities of the transfected cell extracts are presented as relative light units (RLU) and expressed as the mean ± standard deviation of three transfected cultures.

### Immunoprecipitation and western blot analysis

Cells were harvested in lysis buffer (50 mM Tris (pH 8.0), 5 mM NaCl, 0.5% NP-40 and 1x protease inhibitor) and freeze/thawed three times, and the protein was recovered. Protein concentrations were determined using the Bradford method (Bio-Rad, CA, USA). Cell extracts containing equivalent amounts of protein were immunoprecipitated in lysis buffer containing the indicated monoclonal antibody against p53 overnight (4°C). Protein A/G Sepharose beads were added to the immunoprecipitation mixture for 1 hr before three washes with SNNTE buffer (5% sucrose, 1% NP-40, 0.5 M NaCl, 50 mM Tris (pH 7.4) and 5 mM EDTA). The entire immunoprecipitate was then suspended in sodium dodecyl sulfate-polyacrylamide gel electrophoresis (SDS-PAGE) sample buffer, boiled, and loaded onto an SDS-polyacrylamide gel. The separated proteins were transferred onto polyvinylidine difluoride membranes (Millipore, USA) and detected using antibodies against HA (3 F10, Hoffmann-La Roche, Switzerland), p53 conformation (Pab246, Calbiochem, USA) and p53N, p53C, p21 and actin (Santa Cruz Biotechnology, USA).

### Protein-protein interaction analysis

For the GST pull-down assays, ^35^S-labeled proteins were produced with the TNT T7-coupled reticulocyte lysate system (Promega, USA), and GST fusion proteins were expressed in *Escherichia coli* BL21. Radioactively labeled ER or TR proteins were translated *in vitro*, incubated with various immobilized GST-p53 fusion proteins, and eluted and analyzed using SDS-PAGE as previously described [14].

For the co-immunoprecipitation assay, cell extracts containing equivalent amounts of protein were immunoprecipitated in lysis buffer containing the indicated monoclonal antibody against Gal4DBD overnight (4°C). Protein A/G Sepharose beads were added to the immunoprecipitation mixture for 1 hr before three washes with SNNTE buffer. The entire immunoprecipitate was then suspended in SDS-PAGE sample buffer, boiled, and loaded onto an 10% SDS-PAGE. The separated proteins were transferred onto polyvinylidine difluoride membranes (Millipore, USA) and detected using antibodies against HA (3 F10, Hoffmann-La Roche, Switzerland) and Gal4DBD (Santa Cruz Biotechnology, USA).

### Reverse transcription-polymerase chain reaction (RT-PCR) and quantitative real-time PCR

Total RNA was isolated using the TRIsure (BIOLINE, UK) reagent according to the manufacturer’s instructions. One microgram of total RNA was subjected to reverse transcription using MMLV reverse transcriptase for 60 min at 37°C (Epicentre Biotechnologies, USA), and the reactions were run on a GeneAmp PCR system 9700 (Applied Biosystems, USA). The following primers were used for RT-PCR: *p53* forward: *5′-cagtctgggacagccaagtc-3′* and reverse: *5′-cttctgtacggcggtctctc-3′*; *p21* forward: *5′-gagagc*ggcggcagacaacagg*-3′* and reverse: *5′-gcgcccaatacgaccaaatc-3′*; *GAPDH* forward: *5′-agccaaaagggtcatcatctc-3′* and reverse: *5′-gtccaccaccctgttgctgtag-3′*.

## Results

### Structural characterization of the new TP53 variant

**Figure 1:**
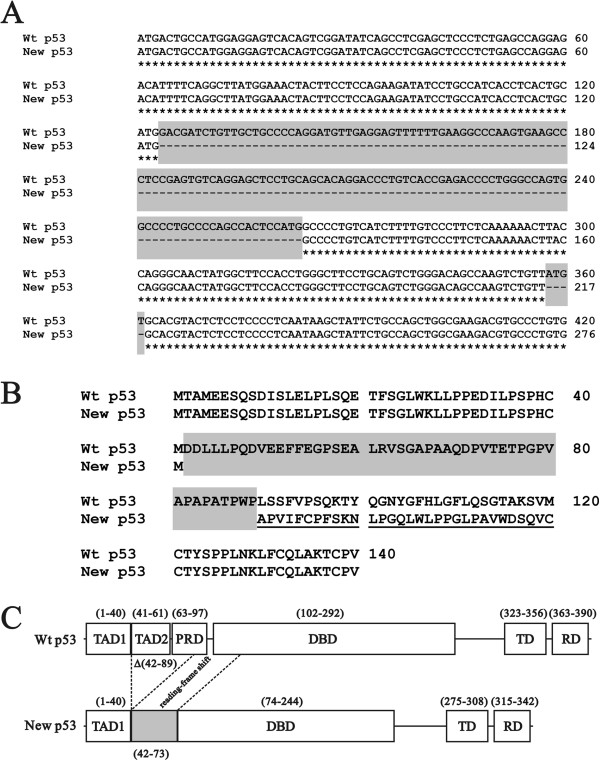
**The sequence information for the new p53 variant. (A)** Two deleted regions (140 nucleotides and four nucleotides), compared with the wild-type p53 mRNA, are labeled in gray. **(B)** Two deleted regions that result in a 48-amino acid deletion (gray area) and 31-amino acid frame-shift (underlined). **(C)** The differences in the functional domains of the new p53 variant are one deletion (Δ42-89) and one reading frame-shift (amino acids 90-120). Other important functional domains, containing TAD1, C-terminal DBD, TD and RD, were intact.

We unexpectedly isolated a novel mouse *TP53* variant (accession number KF766124) containing two deleted regions (one was 140 nucleotides and the other was four nucleotides) in its amino terminus during the regular construction process of wild-type mouse *TP53* (Figure 1A, gray boxes). This new mouse p53 variant expresses one deleted amino acid fragment (from amino acid residues 42 to 89) (Figure 1B, gray boxes) and one reading frame-shift region (from amino acid residues 90-120) (Figure 1B, underlined). Hence, it remains a so-called transactivation domain 1 (TAD 1; amino acid residues 1-40) with most of the DBD (amino acid residues 121-292) and intact C-terminal tetramerization and regulatory domains (TD and RD; amino acid residues 293-390). The difference between this variant and wild-type mouse p53 is shown in Figure 1C, including TAD2, PRD and the N-terminal region of DBD. One unidentified functional region is shown in a gray box.

**Figure 2:**
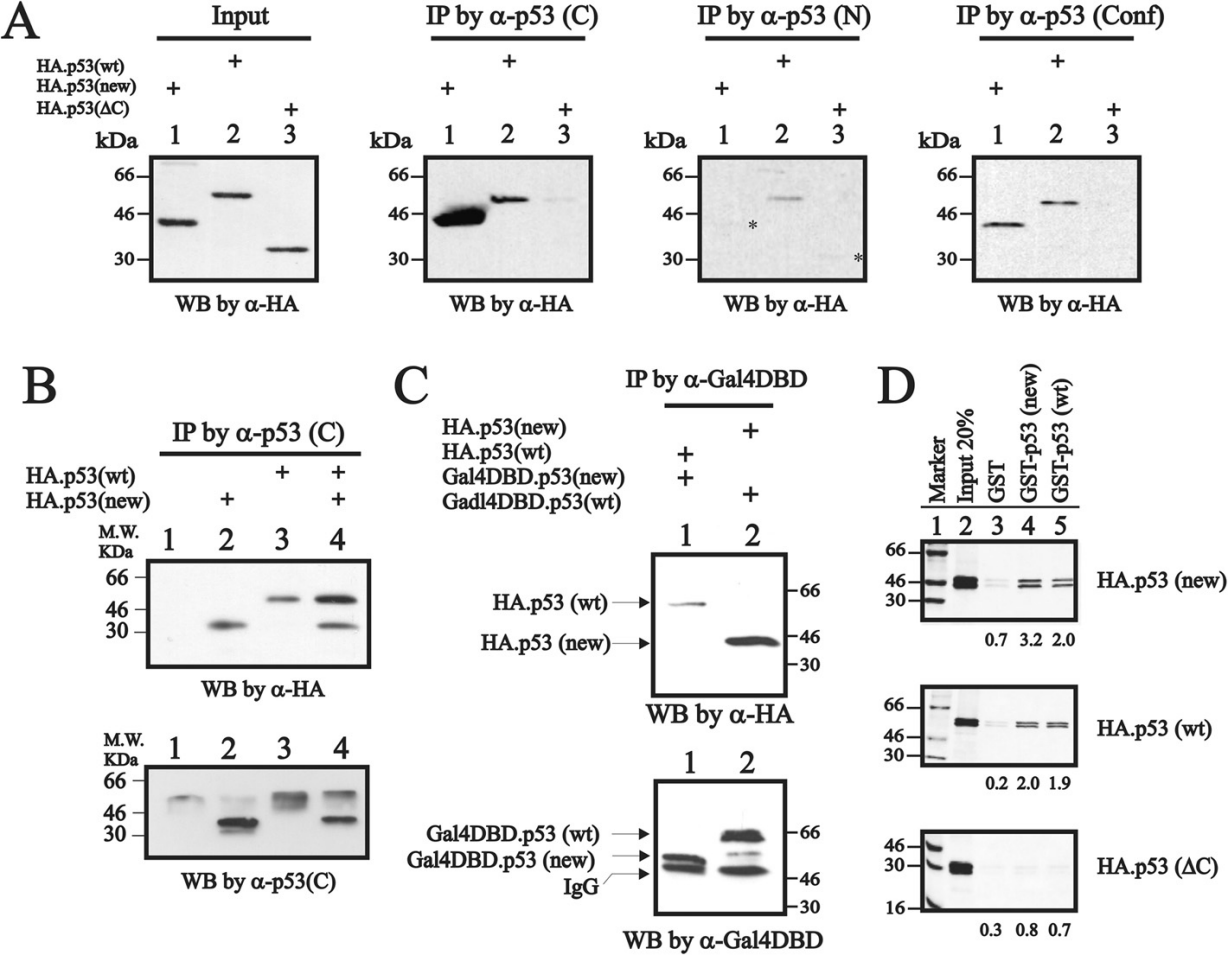
**Protein structures verified using various p53 antibodies. (A)** HeLa cells were transiently transfected with the indicated wild-type, new variant or C-truncated pSG5.HA.p53 expression DNAs (0.8 μg). Various p53 proteins were immunoprecipitated with the indicated antibodies specifically against the C-terminal (C), N-terminal (N) and conformation (Conf) regions. Immunoprecipitated extracts were subject to the western blotting analysis using the HA-tag antibody. **(B)** HeLa cells were transiently transfected with the indicated wild-type or new variant pSG5.HA.p53 expression DNAs (0.8 μg), and 36 hours after transfection, the immunoprecipitated lysates, by specificity for the p53 C-terminus, were subjected to the western blotting analysis using HA-tag and C-terminal antibodies. **(C)** HeLa cells were transiently transfected with the indicated wild-type or new variant pSG5.HA.p53 or/and Gal4DBD.p53 expression DNAs (0.5 μg), and 36 hours after transfection, the immunoprecipitated lysates, by specificity for Gal4DBD, were subjected to the western blotting analysis using HA-tag and Gal4DBD antibodies. **(D)** The indicated wild-type, new variant and C-terminal truncated pSG5.HA.p53 were translated *in vitro* and incubated with bead-bound GST protein. The various GST–p53 (wild-type and new variant) fusion proteins are indicated. Bound proteins were eluted, separated by SDS–PAGE, and visualized by autoradiography. For comparison, the leftmost lane of each panel shows 20% of the input protein used in the binding assay reactions. We observed a similar expression pattern in three independent experiments.

To investigate the structural characterization of this variant compared with wild-type p53, we used various p53 antibodies specifically against p53’s C-terminus (labeled as C), N-terminus (labeled as N) and conformation statuses (labeled as conf) for analyses. The conformation antibody (Pab246) for recognizing wild-type p53 protein in its native conformation, not mutant or denatured p53 protein [16] was employed. Our data suggest that this variant is primarily detectable with antibodies against p53’s C-terminal (C) and conformation (conf) status and is weakly detected by p53 antibodies against its N-terminal (N) region (Figure 2A). Because p53 could form homotetramers using its C-terminal region, we used an antibody directly against p53’s C-terminal region to immunoprecipitate the wild-type, new variant, both combinations and endogenous p53, and found that the new variant was able to form complexes with either wild-type or endogenous p53 (Figure 2B). Furthermore, we used two different tags (HA and Gal4DBD) and a GST pull-down analysis to investigate the relationships between wild-type and the new p53 variant under co-expression conditions. Based on the co-immunoprecipitation and GST pull-down results, it was demonstrated that this new variant had the ability to form a hetero-oligomerization with wild-type p53 (Gal4DBD.new p53/HA.wt p53 or HA.new p53/Gal4DBD.wt p53 complex) via the immunoprecipitation assay with one Gal4DBD antibody (Figure 2C) and the interactions were mediated through the common C-terminal TD of both p53 proteins (Figure 2D).

**Figure 3:**
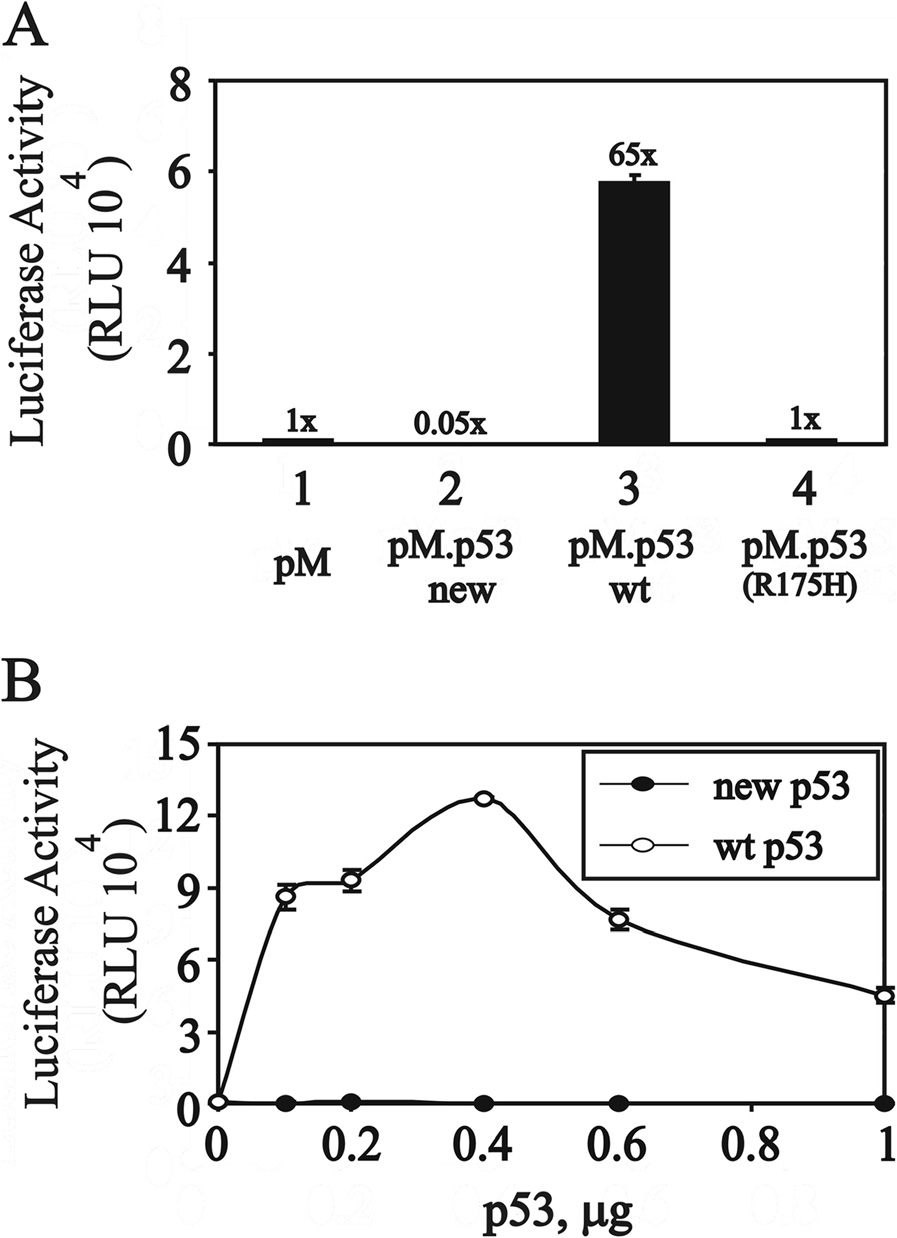
**The new p53 variant has significantly less transactivation activity. (A)** HeLa cells were transiently transfected with 0.4 μg pM (Gal4DBD) and the indicated wild-type, new variant or R175H mutant pM–p53 along with the GK1 reporter gene (0.2 μg). The luciferase activities of the transfected cell extracts were determined. Numbers above the bars indicate the fold increase in activation compared with the pM alone. **(B)** HeLa cells were transiently transfected with the indicated amount of wild-type or new variant pM–p53 along with the GK1 reporter gene (0.2 μg). These data are the average of three experiments (mean ± S.D.; n = 3).

The primary difference between wild-type p53 and the new p53 variant was identified in the PRD and N-terminal DBD (Figure 1C). Many studies have demonstrated that the transactivation domain of p53 is located from amino acid residues 1-40. Unexpectedly, even though our new p53 variant retains an intact TAD1 (amino acids 1-40 fragment), it had no transactivation activity (Figure 3A, compare histograms 2 and 3). Compared with wild-type p53, the new p53 variant had no transactivation activity at all the tested dosages (Figure 3B). However, compared to the control vector (pM), the new p53 variant expressed negative activity, suggesting it might have a repressive effect on the Gal4 DBD luciferase reporter activity (Figure 3A, compare histograms 1 and 2). One hot-spot p53 mutant, R175H, induces structural distortions in the protein and prevents it from binding zinc [11], which also had no transactivation in our system (Figure 3A, compare histograms 4 and 1).

### The functional roles of the new p53 variant in p21 promoter activity and nuclear receptor-dependent repressor activities

**Figure 4:**
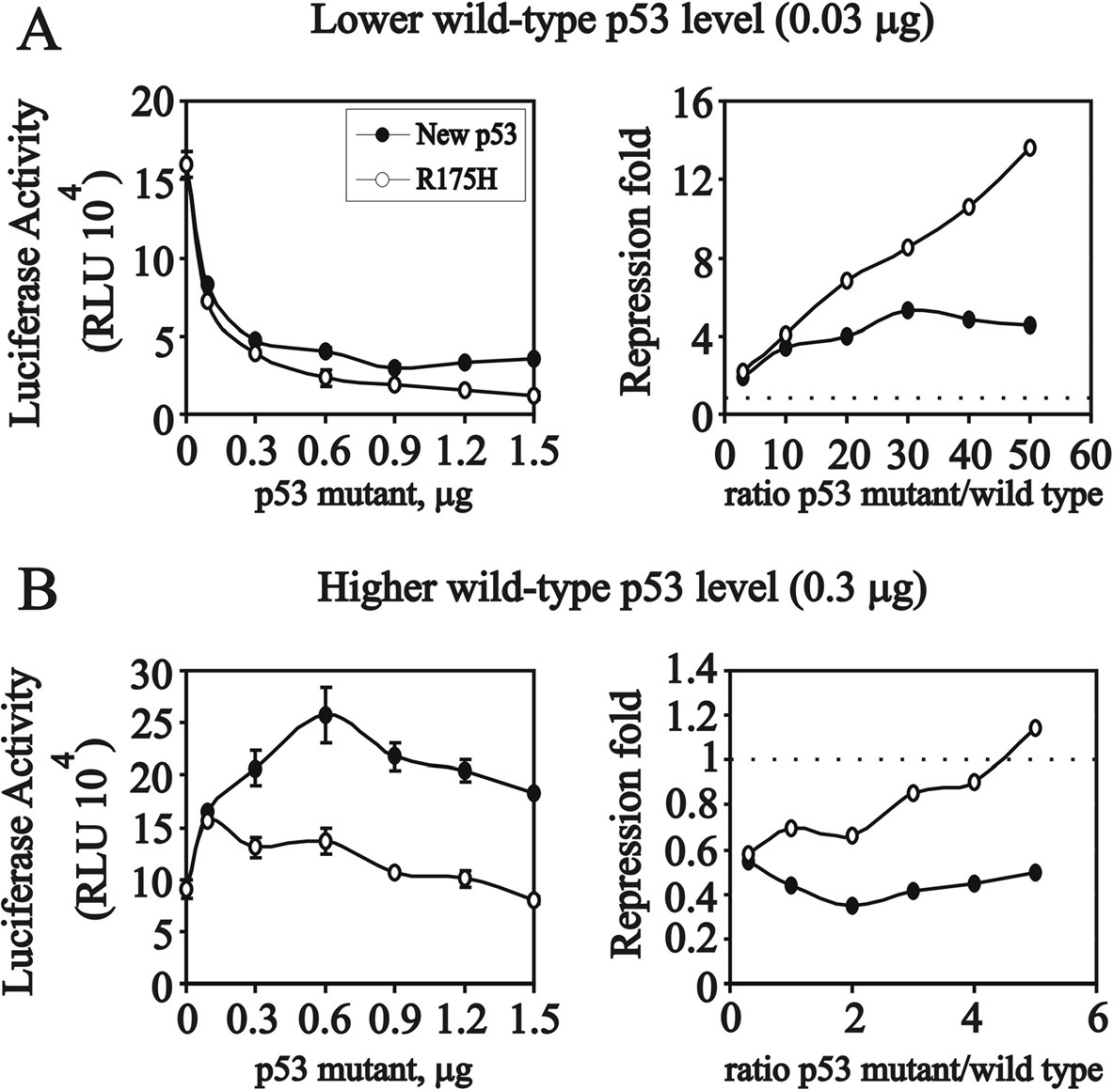
**The endogenous wild-type p53 level affects exogenous p53 functions.** HeLa cells were transiently transfected with 0.03 μg **(A)** or 0.3 μg **(B)** pSG5.HA.p53 and the indicated amount of the new variant or R175H mutant p53 along with the pG13-LUC reporter gene (0.2 μg). The luciferase activities of the transfected cell extracts were determined. These data are the average of three experiments (mean ± S.D.; n = 3).

Based on the hetero-oligomerization and no transactivation activity of the new p53 variant (Figures 1 and 2), we predicted that the new p53 variant might have a dominant-negative effect on wild-type p53 functions, including p53 target gene *p21* regulation and its repressive effect on nuclear receptor (estrogen receptor, ER, and thyroid receptor, TR) activities. We first examined the effects of the new p53 variant and R175H mutant on p53-dependent *p21* promoter activity in the presence of various wild-type p53 levels (ratio of the mutant and wild-type p53). At a lower level (0.03 μg) of wild-type p53, various ratios of the new p53 variant and R175H mutant had repressive effects on *p21* promoter activity (Figure 4A). At a higher level (0.3 μg) of wild-type p53, only the highest ratio of the R175H mutant had a repressive effect on *p21* promoter activity (Figure 4B). Because exogenous introduction of higher amounts of wild-type p53 proteins lowers p21 expression in HeLa cells [17], the new p53 variant seemed to have positive effects on wild-type p53 functions.

**Figure 5:**
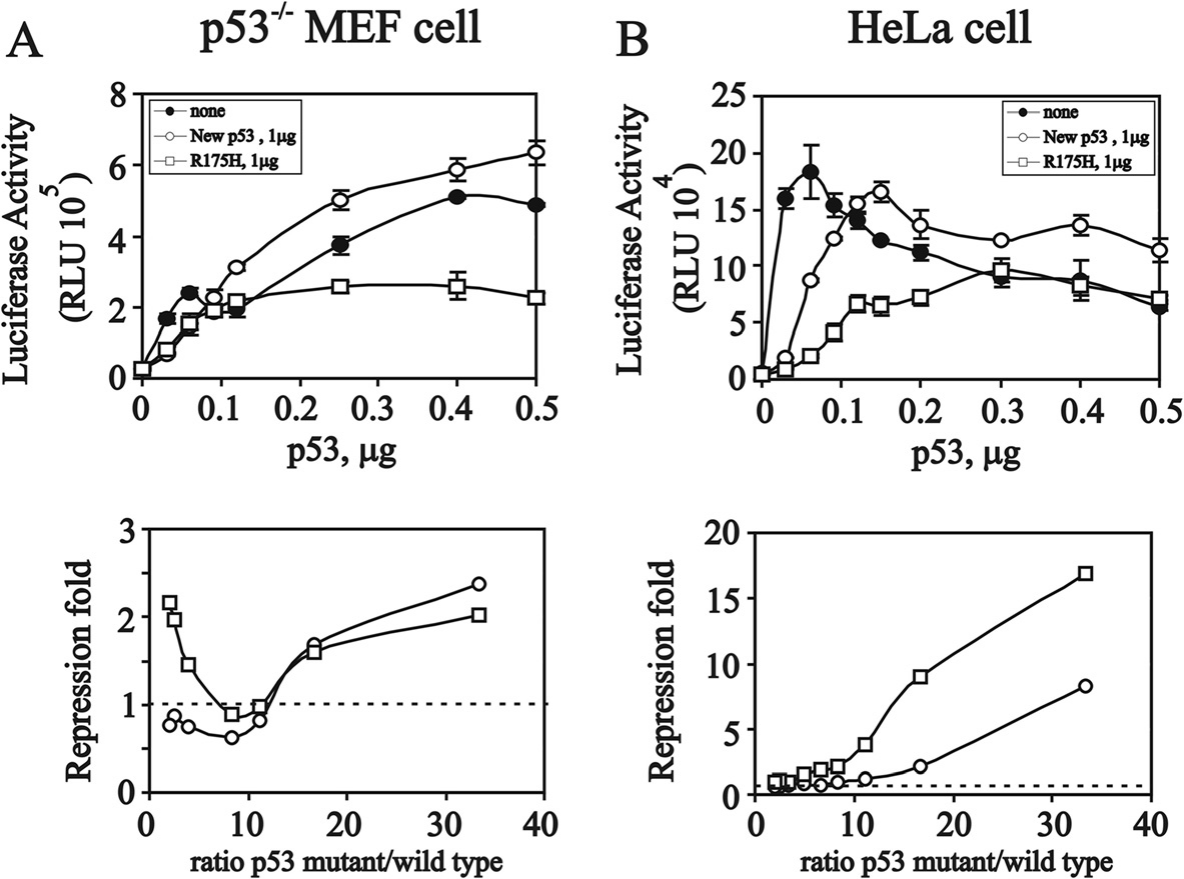
**The functional role of the new p53 variant depends on the endogenous wild-type p53 abundance.** p53^-/-^ MEF **(A)** and HeLa **(B)** cells were transiently transfected with the indicated amount of pSG5.HA.p53, new variant or R175H mutant p53 along with the pG13-LUC reporter gene (0.2 μg). The luciferase activities of the transfected cell extracts were determined. These data are the average of three experiments (mean ± S.D.; n = 3).

Our previous studies have demonstrated that the effect of p53 on the *p21* promoter activity is dependent on the p53 level and that higher amounts of p53 suppress p53-induced activities [13,17]. Next, in mouse embryonic fibroblast (MEF) cells deleted for endogenous p53, higher amounts of the p53 variant failed to suppress wild-type p53-induced activities (Figure 5A, upper panel). Lower and higher ratios of the R175 mutant to wild-type p53 suppressed wild-type p53-induced *p21* promoter activities (Figure 5A, bottom panel, squares). In contrast, the new p53 variant suppressed wild-type p53-induced *p21* promoter activities, and higher ratios of the new p53 variant to wild-type p53 were found (Figure 5A, bottom panel, circles). In HeLa cells, the suppression profiles of the new p53 variant and p53 (R175) mutant were different. The R175 mutant suppressed wild-type p53-induced *p21* promoter activities at all ratios, whereas the new p53 variant only displayed suppression at higher ratios (Figure 5B). In HeLa cells, a higher dose of wild-type p53 suppressed its self-induction [17].

**Figure 6:**
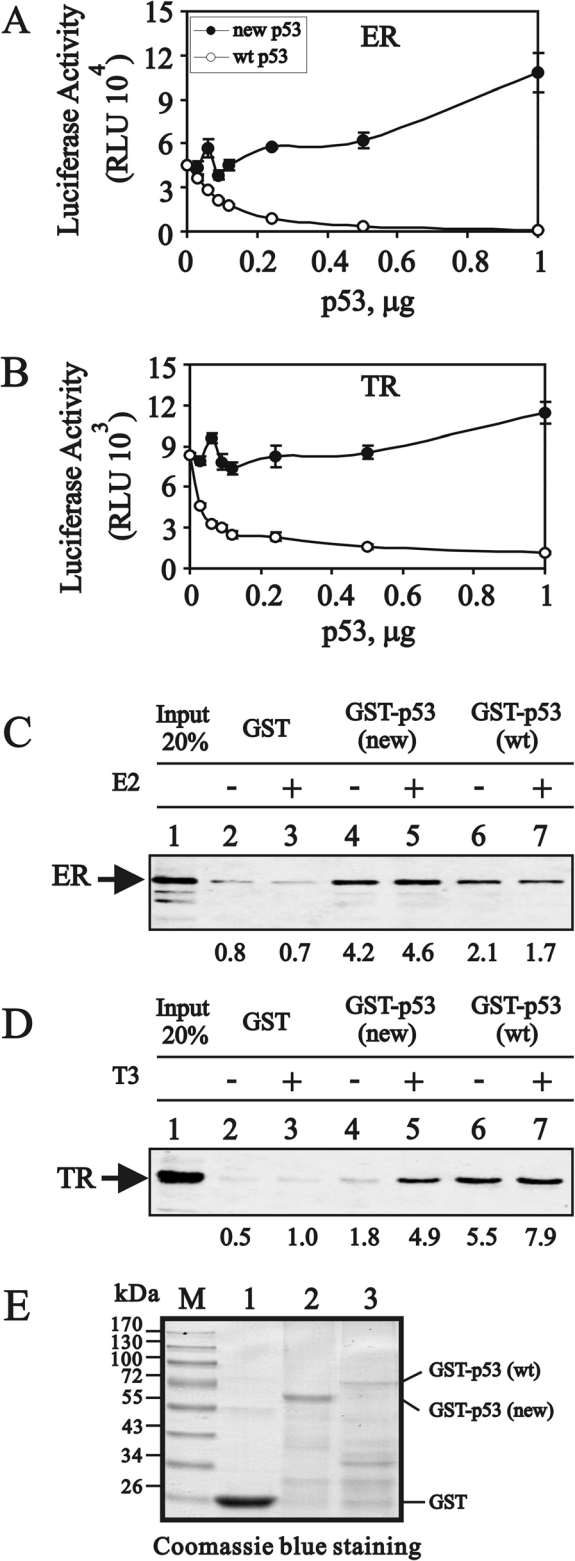
**The new p53 variant binds to ER and TR as well as wild-type p53, whereas it loses the repression on ER and TR transcriptional activities. (A and B)** HeLa cells were transiently transfected with the indicated amount of the pSG5.HA.p53 expression vector; MMTV(ERE)-LUC reporter gene (0.15 μg), pHE0 (0.004 μg) encoding hERα; MMTV(TRE)-LUC reporter gene (0.15 μg), pCMX-hTRβ1 (0.004 μg) encoding hTRβ1. Transfected cultures were grown in **(A)** 100 nM estradiol or **(B)** in 100 nM T_3_, and the luciferase activities of the transfected cell extracts were determined. These data are the average of three experiments (mean ± S.D.; n = 3). **(C and D)** The proteins indicated at the left of each panel (ER and TR) were translated *in vitro* and incubated with bead-bound GST fusion proteins (indicated at the top of each panel along with the indicated p53 fused to GST). The bound proteins were eluted, separated by SDS-PAGE, and visualized by autoradiography. The percentage of labeled protein bound, as determined by phosphorimager analysis, is shown below each lane. For comparison, the left lane of each panel shows the indicated percentage of the input protein used in the binding reaction. The hormone for ER, 100 nM E2, and for TR, 1 μM T3, were included in panels **(C)** and **(D)** (labeled +). We observed a similar expression pattern in two independent experiments. **(E)** Eluted GST and GST–p53 fusion proteins were analyzed by SDS–PAGE and Coomassie blue staining.

Finally, we compared the repressive effects of the new p53 variant on ER- and TR-dependent transcriptional activations with wild-type p53 [18,19]. Wild-type p53 suppressed both the ER and TR activities in a dose-dependent manner (Figure 6A and B, open circles), whereas the new p53 variant enhanced both activities (Figure 6A and B, closed circles). In the GST pull-down analysis, the new p53 variant as well as wild-type p53 directly interacted with ER and TR (Figure 6C and D). The addition of T3 further enhanced the binding of this variant with TR (Figure 6D). Moreover, compared with the amount of GST fusion wild-type p53 proteins used in the pull-down analysis, the new p53 variant might not bind preferentially to ER or TR proteins (Figure 6E).

### Investigating if the new p53 variant is a gain-of-function mutation using tumor cells

**Figure 7:**
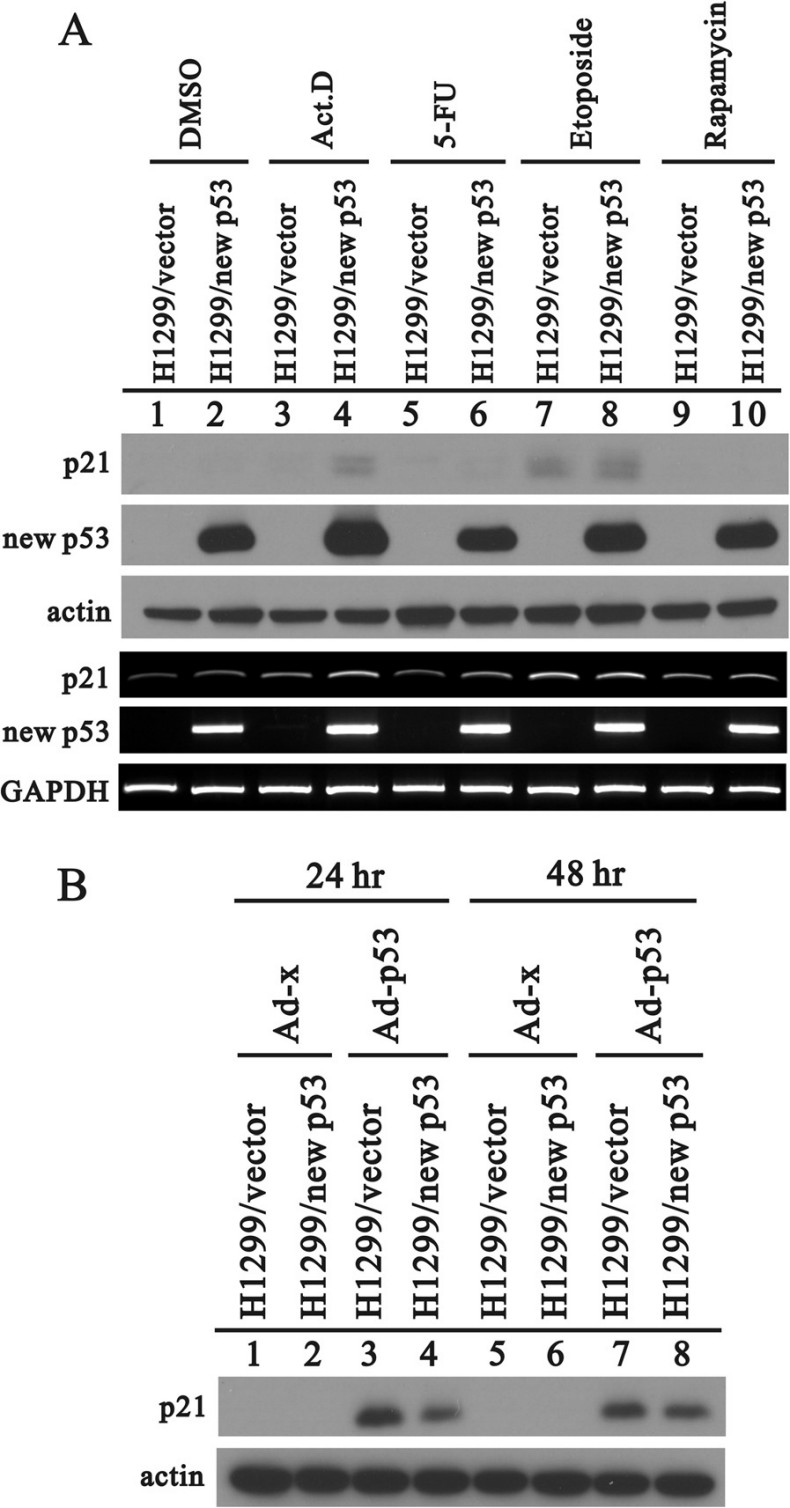
**The gain-of-function of the new p53 variant in tumor cells. (A)** H1299/vector and H1299/new p53 stable cell lines were treated with DMSO (control), Act D (30 nM), 5-Fu, etoposide or rapamycin for 48 h. Protein and mRNA were extracted and analyzed by western blotting and RT-PCR, respectively. **(B)** H1299/vector and H1299/new p53 cells were infected with adenoviral p53 and control. After 24 and 48 h, proteins were extracted and analyzed by western blotting analysis.

To investigate the possibility that the new p53 variant was a gain-of-function mutation, we stably expressed it in H1299 (p53 null) cell lines to examine *p21* gene and protein expression using DNA-damaging drugs such as actinomycin D (Act D), 5-flurouracil (5-FU), etoposide and rapamycin. Without the DNA-damaging insult, the new p53 variant had no inductive effect on *p21* gene or protein expression (Figure 7A). Act D was the only tested DNA-damaging drug to induce *p21* gene and protein expression in the presence of the overexpression of the new p53 variant (Figure 7A). Overexpressing wild-type p53 induced p21 protein expression in H1299 cells (Figure 7B), which could be suppressed by the stable expression of the new p53 variant. Overexpressing various amounts of the new p53 variant in HeLa cells was used for the cell cycle profile. A significant reduction of the subG1 population switching into G2/M phase was observed (data not shown).

**Figure 8:**
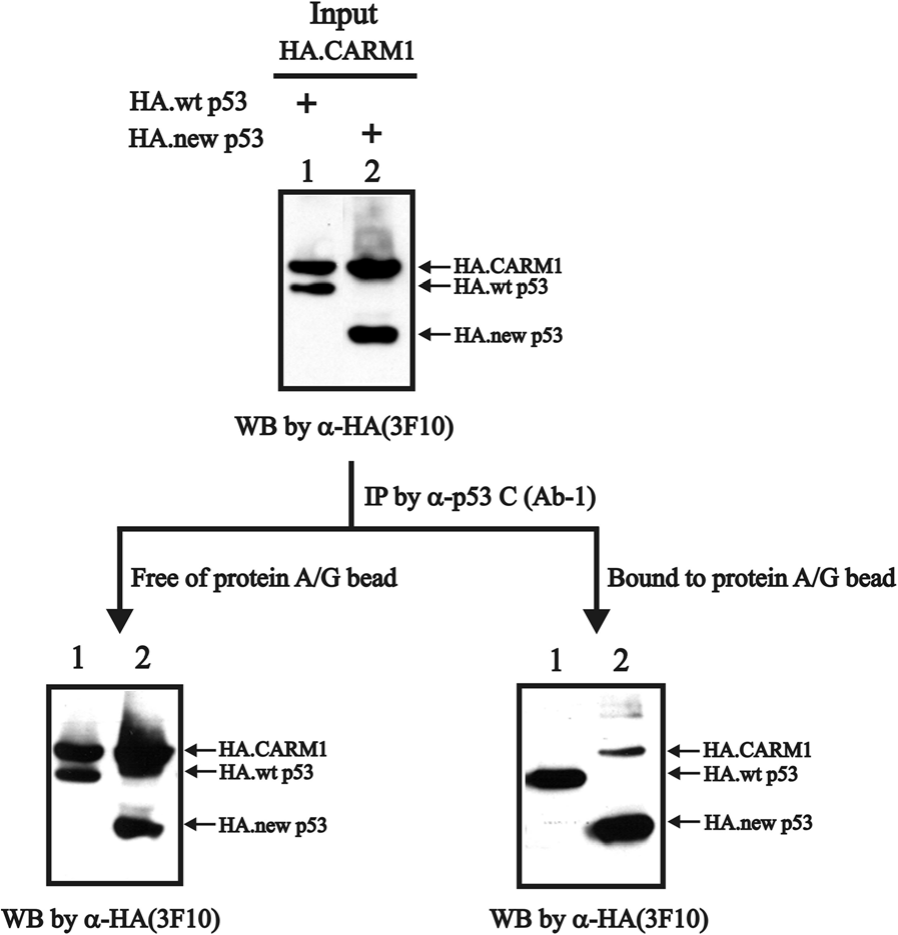
**The new p53 variant, not wild-type p53, complexes with the arginine methyltransferase CARM1 in HeLa cells.** HeLa cells were transiently transfected with 0.5 μg of the indicated pSG5.HA.p53 or new p53 variant along with 0.5 μg of pSG5.HA.CARM1. After 36 h of transfection, cell lysates were immunoprecipitated using an antibody against the p53 C-terminal region and then separated using protein A/G beads. Supernatant (left panel) or protein-bound A/G beads (pellet, right panel) were subjected to immunoblotting analysis using an antibody against HA (3 F10). We observed a similar expression pattern in three independent experiments.

A previous study demonstrated that p300 and CARM1 (co-activator associated arginine methyltransferase 1) exhibit orderly cooperative functions on p53-dependent functions, which are mediated through physical interactions with p53’s N-terminal and C-terminal regions, respectively [20]. CARM1 was originally identified to be functionally linked to nuclear receptor-dependent transcriptional regulation [21]. CARM1 regulates a number of additional cellular processes, including cell cycle progression and the DNA damage response [22-24]. Hence, we examined the physical interaction between CARM1 with wild-type p53 or the new p53 variant using the co-IP experiment. Our findings suggest that only the new p53 variant had the ability to associate with CARM1 in HeLa cells (Figure 8).

## Discussion

Because only one p53 isoform, p53/47, could be generated by alternative splicing, representing an approximately 2 × 10^20^ lower amount of p53/47 mRNA [25], our new p53 variant should also be a rare event as the full-length p53 mRNA was mediated through at least two unidentified alternative splicing pathways. The detailed regulatory mechanism remains to be investigated in the future. Currently, p53 mutations can generally be classified as either “conformational” or “DNA contact” mutants [11]. Mutations of the p53 gene represent the most frequent genetic alterations in human cancers, affecting approximately 50% of all individual tumors. The major effect of these mutations is the elimination of various wild-type p53 tumor-suppressing functions, including apoptosis and growth arrest. Two consequences are selected for by the accumulation of p53 mutations in tumor cells: i) a dominant-negative role by hetero-oligomerization with wild-type p53 expressed by the second allele, or ii) a specific gain-of-function of mutant p53. The TP53 Mutant Loss Of Activity Database provides 26 different TP53 activities, covering biochemical activity, structure, biological activity and gain-of-function, to identify the functions of target p53 mutants (http://p53.fr/TP53Mutload/TP53Mutload.html). It is still unclear as to whether our reported p53 variant should be defined as a p53 isoform or as a mutant. Our new p53 variant is not a single amino acid mutant but is rather one 48-amino acid fragment deletion followed by one 31-amino acid reading frame-shift fragment. It has intact N-terminal (amino acids 1-41) and C-terminal (amino acids 121-393; DNA binding, tetramerization and regulatory domains) regions, suggesting that it serves as a dominant-negative mutant. Our presented data also support this property by the fact of hetero-oligomerization with wild-type p53 through its intact conformation and no transactivation activity. Generally, the more the mutation disrupts the original wild-type conformation, the less wild-type p53 activity will be retained, and the more likely it is that the new oncogenic functions will prevail. Our study appears to fail to support this trend because of the intact conformation and lack of transactivation activity. Combined with our current findings, this new p53 variant seemingly does not fit the definition of a “conformational” or “DNA contact” p53 mutation.

A transactivation activity as well as a specific sequence-binding ability is important for a transcriptional factor, including p53. Here, we report that a new p53 variant has lost its transactivation activity. Many studies indicate that p53 has two distinct TADs, 1 and 2 [26,27]. Based on this definition, our new p53 variant at least retains an intact TAD 1 even though it has a lower than basal Gal4 transactivation activity (Figure 3A). There are at least two possibilities for our findings. One is the importance of the deleted region (amino acid residues 42-89 of wild-type p53), which has been identified as the TAD (covering amino acid residues 1 to 58), and the other is the repressive role of the reading frame-shift region (amino acid residues 42-73 of the new p53 variant) (Figure 1C). Our previous results demonstrated that the fusion of amino acids 1-40 of p53 with Gal4DBD expresses a better luciferase activity compared to full-length p53 [13]. Hence, the repressive role of the reading frame-shift region might be the reason why our new p53 variant loses its total transactivation activity.

Here, our reported p53 variant was not able to regulate p21 or other target genes because of the loss of its transactivation activity. Our data reveal that its intact C-terminal region has the ability to hetero-oligomerize with wild-type p53 for a dominant-negative effect. Our data demonstrate that this new p53 variant could suppress the activation or repression effect by wild-type p53. p53 mutants may lose certain tumor-suppressive functions of wild-type p53 while retaining and/or exaggerating other aspects of normal wild-type p53 function. The work of Di Agostino et al. (2006) demonstrates that in response to treatment with adriamycin, wild-type p53 and mutant p53 recruit different transcriptional cofactors: the histone deacetylase HDAC1 in the case of wild-type p53 and the histone acetyltransferase p300 in the case of mutant p53 [28].

We further examined the possibility of a gain-of-function for our reported p53 variant. While the gain-of-function concept of mutant p53 is well established, the exact criteria for how it works can still be quite confusing [29]. Two primary mechanisms are commonly proposed to address such events: (1) an interaction between mutant p53 and cellular proteins, or (2) the mutant p53-mediated regulation of novel target genes. Given a previous study suggests that wild-type p53 interacts with PRMT5, not CARM1 [30], and the suppression of HPV E6 proteins by the arginine methyltransferase activity of CARM1 might be reasoned for why wild-type p53 fails to physically interact with CARM1 in HeLa cells [31], our current data however showed that the discovered new p53 variant interacts better with CARM1 (Figure 8). As a result, the gain-of-function of the new p53 variant has the potential to modulate the post-translational modification activity of CARM1 on arginine methylation of its target proteins, such as histone H3 and other non-histone proteins [20,21,24,30]. In this regard, although we did not comprehensively assess p53-related specific gene regulations or cell cycle arrest in responding to DNA damage [20,24], we cannot rule out the possibility that the new p53 variant regulates novel target genes through its binding to specific sequence element(s). Thus, it is believed that this new p53 variant might attribute to modulate many CARM1-modified proteins via the protein-protein interaction *in vivo.*

## Conclusions

Taken together, our findings provide valuable information about the structure and function of p53 for the regulation of transactivation activity and cellular protein-protein interactions. Furthermore, natural p53 isoforms will help us understand the functional roles of the p53 family and potential therapeutics for p53-dependent cancers.

## Competing interests

The authors declare that they have no competing interests related to this work.

## Authors’ contributions

SMH carried out the sequence analysis of new p53 variant. JYHC carried out the immunoprecipitation, co-immunoprecipitation and reporter analysis. YCC carried out the gain-of-functions. STL participated in the GST pull-down analysis and reporter analysis. WYC, CLH and SMH conceived of the study, and participated in its design and coordination and helped to draft the manuscript. All authors read and approved the final manuscript.
